# Temporal Changes in Lead and Cadmium Levels in *Amanita muscaria* Samples Collected in Poland

**DOI:** 10.3390/toxics13020101

**Published:** 2025-01-27

**Authors:** Michal Ordak, Aleksandra Galazka, Pawel Konieczynski, Marek Wesolowski, Alina Plenis, Elzbieta Muszynska, Przemyslaw Kurowski, Magdalena Bujalska-Zadrozny

**Affiliations:** 1 Department of Pharmacotherapy and Pharmaceutical Care, Medical University of Warsaw, 02-091 Warsaw, Poland; s080868@student.wum.edu.pl (A.G.); przemyslaw.kurowski@wum.edu.pl (P.K.); magdalena.bujalska@wum.edu.pl (M.B.-Z.); 2 Department of Analytical Chemistry, Medical University of Gdansk, 80-416 Gdansk, Poland; pawel.konieczynski@gumed.edu.pl (P.K.); marwes@gumed.edu.pl (M.W.); alina.plenis@gumed.edu.pl (A.P.); 3 Department of Medical Biology, Medical University of Bialystok, 15-222 Bialystok, Poland; elzbieta.muszynska@umb.edu.pl

**Keywords:** *Amanita muscaria*, cadmium, lead

## Abstract

In recent years, an increasing popularity of consuming *Amanita muscaria* has been observed in Poland, aimed at reducing various medical symptoms. However, there is a lack of data regarding the impact of variations in the content of toxic elements, such as cadmium (Cd) and lead (Pb), in *Amanita muscaria* collected during late summer and mid-fall. The aim of this study was to determine the concentrations of Pb and Cd in *Amanita muscaria* samples collected at different times of the year, compare the concentrations of these elements in samples with and without cap skin, and compare the obtained values to permissible limits in dietary supplements. A total of 44 *Amanita muscaria* samples were collected during three different harvesting periods (August, September, and October 2023) from Puszcza Biała, located approximately 80 km from Warsaw. The mushrooms were subjected to mineralization using concentrated nitric acid and hydrogen peroxide, followed by the determination of Pb and Cd concentrations using an atomic absorption spectrometer. Significant statistical differences were found in the Pb concentrations of samples collected in three different seasons (η^2^ = 0.67, *p* < 0.001), with the concentration increasing progressively, reaching its highest value in October. Similarly, the Cd concentration also increased in the later collections, although the effect of time was weaker (η^2^ = 0.13, *p* = 0.03). No significant differences were observed in Pb and Cd concentrations between samples with and without cap skin. The average Cd concentrations in mushrooms were significantly higher than the permissible levels in dietary supplements; they were four times higher in August (*p* < 0.001), six times higher in September (*p* < 0.001), and nine times higher in October (*p* < 0.001). The Pb concentration in the October samples was close to the permissible limit but did not exceed it in a statistically significant manner (*p* = 0.85). Due to the high Cd concentrations and potentially hazardous levels of Pb, the consumption of *Amanita muscaria* carries a significant risk of toxicity, which may lead to serious health hazards, particularly in the context of prolonged exposure.

## 1. Introduction

Currently, Europe is experiencing a rapidly growing drug market. There has also been a sharp increase in the number of new psychoactive substances (NPS) [1]. These types of chemical substances are highly pure and potent, which can lead to an increased number of fatal incidents. In 2017, there were 8238 reported deaths due to overdoses [2], while during the COVID-19 pandemic, 130 deaths were attributed solely to NPS [3]. Due to active efforts in combating drugs, obtaining them has become increasingly difficult. In extreme cases, this leads to attempts at the self-synthesis of drugs, especially if a precursor can be easily obtained from any pharmacy without a prescription. One example is ephedrine, produced from medications containing pseudoephedrine [4]. A side effect of the fight against drugs is the increased search for new, easily accessible, and legal psychoactive agents. The latest trend in Poland is the consumption of *Amanita muscaria*. Research results published in 2023 in *Toxics* indicated that an analysis of 5.600 comments allowed for the examination of the reasons for consuming *Amanita muscaria*, forms of ingestion, and reported side effects. Among women, the primary reasons for consumption were pain relief and alleviation of skin issues, whereas, among men, the leading reasons were stress reduction, alleviation of depressive symptoms, and insomnia, with women more often choosing tinctures and men preferring dried forms of the mushroom. These results highlight the need for further research on the toxicity of *Amanita muscaria* to raise public awareness about the risks associated with its consumption [5]. In the same year, results of a study were published in *Studia Paedagogica Ignatiana*, involving 95 participants who consumed *Amanita muscaria* either regularly or occasionally. Participants were divided into two groups: those consuming the mushroom sporadically and those who did so regularly, at least several times per month. About 90% of the respondents consumed the fly agaric in dried form, 52.6% used extracts, and 17.9% consumed it raw. The majority of *Amanita muscaria* enthusiasts collected the mushrooms themselves, with the main reasons for consumption being curiosity, expected medicinal effects, and spiritual needs [6]. Given the increasing frequency of *Amanita muscaria* consumption, it seems justified to analyze the concentrations of toxic elements such as Pb and Cd in this mushroom.

Pb toxicity can lead to severe neurological disorders, kidney damage, cardiovascular issues, and developmental disorders, particularly in children. Pb is neurotoxic and can accumulate in the body, causing chronic poisoning even at low exposure levels. Cd is highly nephrotoxic, leading to kidney damage, osteoporosis, and an increased risk of cancers, especially lung cancer. Long-term exposure to Cd, mainly through contaminated food, can also cause respiratory diseases and liver dysfunction [7,8].

The data published so far on the Pb concentration in *Amanita muscaria* indicate variability in Pb content in *Amanita muscaria* caps depending on the location, suggesting that different collection sites affect the level of Pb accumulation in the mushroom. On the other hand, it was observed that Pb levels in the caps and stems of the mushroom are comparable, suggesting no difference in Pb accumulation between these parts within a single location [9,10]. Unlike Pb, Cd in *Amanita muscaria* shows varying levels of accumulation depending on the part of the mushroom. Typically, Cd concentration is higher in the caps than in the stems, suggesting preferential accumulation in this part of the fruiting body. These differences may result from the larger surface area of the caps being in contact with metals present in the soil or air. Cd accumulation in *Amanita muscaria* also depends on the concentration of this element in the soil, indicating a strong influence of local environmental conditions on its bioconcentration in the mushroom [9,10,11,12,13,14].

However, published research lacks an examination of how the concentrations of these elements in *Amanita muscaria* change over the course of the collection period. Additionally, no data exist on Pb and Cd concentrations in *Amanita muscaria* collected near the Polish capital. Previous studies have primarily focused on regions in other parts of Poland, including the northern and western areas. There is also no data regarding the influence of *Amanita muscaria* cap skin on the concentrations of these two elements.

Therefore, the aim of this article was to determine the concentrations of Pb and Cd in *Amanita muscaria* samples collected during three different seasons from a site in central Poland. The second aim was to compare the concentrations of Pb and Cd in samples of *Amanita muscaria* with and without cap skin, as, based on studies on other mushrooms, its presence may influence the concentrations of toxic metals [15]. An additional goal was to analyze whether the levels of Cd and Pb in the collected material exceeded the permissible concentrations of these elements in dietary supplements.

## 2. Material and Methods

### 2.1. Samples of Amanita muscaria

From a location near Warsaw (approximately 80 km away), forty-four samples of *Amanita muscaria* were collected between August and October 2023. All mushrooms were gathered from Puszcza Biała, Brok, Poland (52° 43′ 7.24″ N, 21° 53′ 44.695″ E). All samples were collected from the same site in Puszcza Biała in August, September, and October. In August, ten samples were collected, of which four were without cap skin and six with cap skin. In September, seventeen mushrooms were collected, eleven with cap skin and six without cap skin. In October, seventeen mushrooms were also collected, with eleven having cap skin and six without. The collection of mushroom samples in August 2023 (25 August 2023) depended on the first appearance of mature fruiting bodies in the forest, considering that August was a dry month, which limited the availability of mushrooms. To maintain a consistent time interval between collections, samples in September (10 September 2023) and October (13 October 2023) were collected approximately one month apart. Simultaneously, during the decision-making process for collection, the availability of mushrooms in the forest was also taken into account, ensuring that samples were collected when they were present in their natural environment. The harvested samples were dried at room temperature in a well-ventilated area to reduce their high moisture content, which could have initiated adverse biological processes. After drying, the mushrooms were secured in paper bags and stored in a dry, shaded place until analysis. The dried samples were cleaned of residual soil using a brush or by cutting off the most contaminated parts, and then the mushroom caps and stems were ground together.

### 2.2. Determination of Lead and Cadmium

A total of 44 mushroom samples were analyzed. Approximately 0.5 ± 0.05 g of the ground mushroom was weighed. To each sample, 10 mL of concentrated HNO_3_ and 1 mL of 30% H_2_O_2_ were added consecutively. The four-step mineralization process (Table 1) was carried out using a Jupiter-Sineo apparatus (Shanghai, China). Before opening the cap, the sample was cooled in air for approximately 10 min. The mineralizates were then quantitatively transferred to a 50 mL volumetric flask, and redistilled water was added to reach the final volume due to its suitability and easy availability for the mineralization process. The samples were analyzed using an atomic absorption spectrometer AAS 250 Plus (Varian, Sydney, Australia) to determine the concentrations of Pb and Cd.

The standard solutions of Cd contained 0.2, 0.4, 0.6, 0.8, and 1.2 µg per 1 mL. Based on these, a calibration curve was created with the following equation: ACd = 0.3026 × CCd − 0.0012; R^2^ = 0.9959. The calibration curve for Pb contained 1, 2, 3, and 4 µg per 1 mL of solution. The curve was linear and described by the following equation: APb = 0.0332 × CPb + 0.008; R^2^ = 0.9937.

The precision and accuracy of the applied FAAS method were determined based on measurements of metallic elements in a Certified Reference Material, specifically the Mixed Polish Herbs (INCT-MPH-2). To check the precision of Pb and Cd measurements, the absorption of two randomly chosen solutions was determined, for which 12 repetitions were performed. The table below presents the results of the precision and accuracy of the method (Table 2). Based on the obtained results, it was concluded that the measurements were accurate and precise.

### 2.3. Statistical Analysis

Each sample was analyzed in triplicate, and the results were presented as the arithmetic mean (M) with standard deviation (SD). The concentration of Pb and Cd in the *Amanita muscaria* samples was expressed in microgram/g dry mass (d.m.) and in ng/g d.m., respectively. Statistical analysis was performed using IBM SPSS Statistics 25. The Mann-Whitney U test was used to check for statistically significant differences between two independent groups of *Amanita muscaria* samples. In the case of more than two groups (harvest periods), the Kruskal-Wallis test was used. When statistically significant differences were observed, Dunn’s post-hoc test was applied to determine the exact nature of these differences and which groups differed from each other. The eta-squared measure was used to quantify the strength of the effect size in the analysis. Values ranging from 0.01 to 0.06 indicate a small effect, 0.06 to 0.14 represent a medium effect, and 0.14 or higher suggest a large effect. The one-sample *t*-test was used to assess whether the concentrations of Cd and Pb differed from the maximum permissible levels of these elements in dietary supplements. In the statistical analysis of the results, the following descriptive statistics were used: mean, median, first quartile, and third quartile. A significance level of *p* < 0.05 was considered statistically significant.

## 3. Results

### 3.1. Temporal Variability in Cd and Pb Concentrations in Amanita muscaria

A strong relationship was observed between the passage of time and the concentration of Pb in the collected *Amanita muscaria* samples. The later the harvest period, the higher the Pb concentration. This applies to both mushrooms without cap skin and those with cap skin. In the case of Cd, a statistically significant relationship was found in all collected mushroom samples, as well as in samples with cap skin, but this relationship was weaker compared to Pb (Table 3).

**Figure 1:**
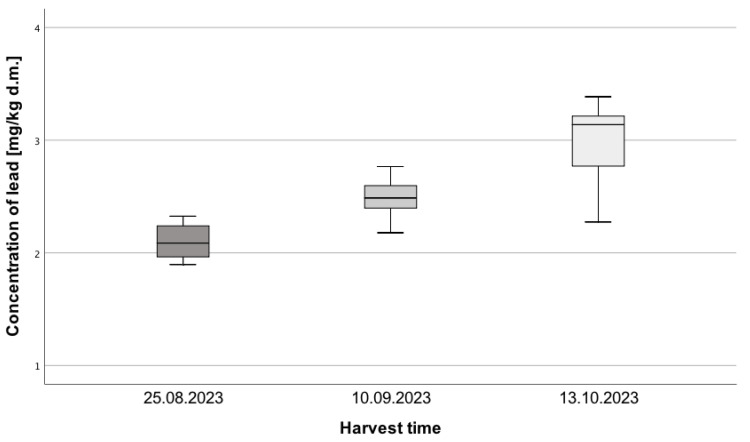
Pb Concentration in *Amanita muscaria* samples collected on three dates: 25 August, 10 September, and 13 October 2023 (N = 10, 17, 17).

The obtained results are confirmed by statistically significant differences in Pb concentration among the different time periods, H = 29.45; *p* < 0.001; η^2^ = 0.67 (Figure 1). Pairwise comparisons using Dunn’s post-hoc test revealed that each harvest period differs significantly from the others in terms of the concentration of this element. In the last harvest period, the concentration of this element was higher compared to the beginning of the collection (*p* < 0.001) and the second period (*p* = 0.003). The same applies to the comparison of the second period to the first (*p* = 0.005). The effect size of 0.64 indicates a significant impact of the passage of time on the Pb concentration in the collected *Amanita muscaria* samples.

**Figure 2:**
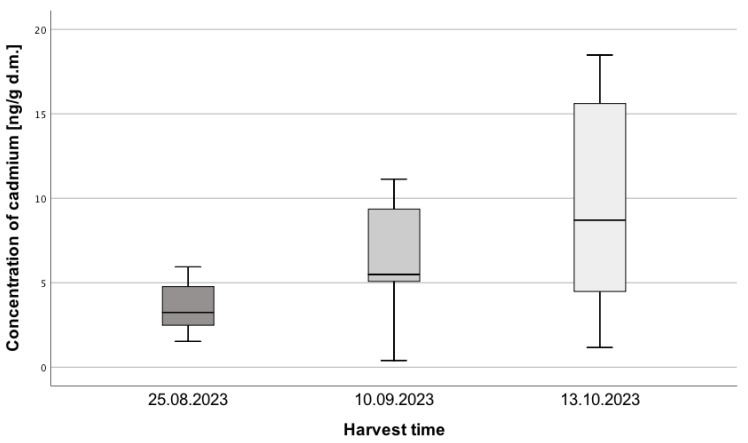
Cd Concentration in *Amanita muscaria* samples collected on three dates: 25 August, 10 September, and 13 October 2023 (N = 10, 17, 17).

Statistically significant differences were also observed in Cd concentrations, H = 6.99; *p* = 0.03; η^2^ = 0.13 (Figure 2). The Cd concentration in the last harvest period of *Amanita muscaria* was higher compared to the first period (*p* = 0.03). The effect size of 0.13 indicates a moderate effect of the passage of time on the Cd concentration in the collected *Amanita muscaria* samples.

Statistically significant differences were also observed in Pb concentrations in samples of *Amanita muscaria* without cap skin (H = 13.24; *p* = 0.001; η^2^ = 0.87) and with cap skin (H = 15.4; *p* < 0.001; η^2^ = 0.54). The pairwise comparison analysis revealed that in the group of mushrooms without cap skin, the Pb concentration at the end of the harvest was higher compared to the second period (*p* = 0.03) and the beginning of the collection (*p* < 0.001). In the case of mushrooms with cap skin, the Pb concentration at the end of the harvest was higher compared to the second period (*p* = 0.03) and the first period (*p* < 0.001). The same applies to the comparison of the second harvest period to the first (*p* = 0.04). No statistically significant differences were found regarding the Cd concentrations. This applies to both samples of *Amanita muscaria* without cap skin (H = 2.88; *p* = 0.24) and with cap skin (H = 4.61; *p* = 0.1).

### 3.2. Influence of Cap Skin Presence on Cd and Pb Concentrations

Additionally, the results of the statistical test regarding the influence of the presence of cap skin on the concentrations of Pb and Cd in the collected *Amanita muscaria* samples are presented in the table below (Table 4). No statistically significant differences were observed.

### 3.3. Permissible Concentrations of Cd and Pb in Dietary Supplements and Their Average Concentrations in the Analyzed Samples

In the next step, it was determined whether the average concentrations of Pb and Cd in the analyzed *Amanita muscaria* samples differ significantly from the permissible concentrations of these elements in dietary supplements [16]. The permissible concentration for Cd is 1 µg/g, while for Pb, it is 3 µg/g. The analysis was conducted by dividing the data into three time periods (Table 5). The results indicated that the average concentration of Cd was significantly higher than the permissible levels of this element in dietary supplements. The Cd concentration was over 4, 6, and 9 times higher in the collected *Amanita muscaria* samples compared to the permissible levels.

In the case of Pb, the concentration obtained was lower than the standard in the first two harvest periods. Regarding the last harvest period, no significant differences were found. However, the average concentration of this element during this period was at the threshold of the permissible norm.

## 4. Discussion

Pb is a heavy metal that can be found in electronic waste, paints, batteries, glazed ceramics, contaminated water, cosmetics, traditional medicines, industrial emissions, and industrial legacy exposure to Pb [17]. It is a harmful element to health, particularly dangerous for children, as it can negatively impact brain development, leading to reduced intelligence quotient (IQ), low birth weight, and increased risk of miscarriage [18]. Cd is a heavy metal that has harmful effects on human health, even at low concentrations, leading to serious diseases and potential fatalities due to its toxic nature [19]. The results published in *Toxics* indicate that proponents of consuming *Amanita muscaria* cite the desire to alleviate stress, pain, and insomnia and to reduce the severity of depressive symptoms as their reasons for consumption [5].

Our study results indicate that the later the harvest of *Amanita muscaria*, the higher the Pb concentration. There is a limited amount of data in the literature regarding how the concentration of this analyzed element changes over specific time periods. In research published in *Environmental Science and Pollution Research*, the impact of seasonal variability on Pb concentrations in the shoots of *Trifolium repens* and *Lolium perenne* was evaluated from autumn 2014 to autumn 2015. The studies showed statistically significant differences between the three sampling periods, with Pb levels being higher in each [20]. Subsequent studies recorded seasonal fluctuations in Pb concentrations in the leaf parts of alpine plants, with values higher in winter months (13.5 µg/g d.m.) and early spring months (19.4 µg/g d.m.) than in autumn months (11.0 µg/g d.m.) [21]. Research published in *Fungal Biology* analyzed the impact of the maturity stage of the mushroom on Pb concentration in *Amanita muscaria*. However, seasonal variability was not considered. The results showed that the concentration decreased at each subsequent stage of development of the red mushroom. Initially, it was 0.11 µg/g dry mass (d.m.) in the caps, but after reaching maturity, it dropped to 0.062 µg/g d.m. The concentration in the stems was 0.13 µg/g d.m. at the beginning of growth and 0.07 µg/g d.m. after growth was completed [9]. In another study, fruiting bodies of *Amanita muscaria* were collected between September and October 2006. The results indicate that the Pb content in the *Amanita muscaria* fruiting bodies was at a level of 530 µg/g d.m., which is likely associated with a high accumulation of this element in the soil [10]. In the case of Cd, published studies to date suggest that the concentration of this metal increases with successive stages of development. This metal is intensely accumulated in the fruiting body [9,10]. The results obtained indicated a significant increase in Cd concentration over time. The later the harvest of *Amanita muscaria*, the higher the Cd concentration. This confirms the results obtained and indicates a clear trend of Cd accumulation in *Amanita muscaria*. Another study found that the concentration of Cd in *Amanita muscaria* ranged from 10 to 21 µg/g dry mass in caps and from 4.2 to 10 µg/g dry mass in stems. However, the lack of precise harvest dates complicates the assessment of seasonal variability [11].

The higher Pb concentration in the *Amanita muscaria* samples collected in autumn may suggest that rainfall promotes the accumulation of this metal in mushrooms through several mechanisms. Firstly, rain may increase the leaching of Pb from the soil, leading to increased availability of this metal for plants, including mushrooms. Moreover, rain may also affect the metabolic activity of mushrooms, enhancing their ability to uptake and accumulate metals from the environment. Additionally, moist conditions favor the decomposition of organic matter in the soil, which may influence the release of Pb and its movement to plants through biogeochemical processes [22,23,24]. The observed higher concentrations of Cd in the *Amanita muscaria* samples collected in the fall suggest that rainfall may lead to the leaching of Cd from the soil. Additionally, rain may stimulate the metabolic activity of fungi, increasing their capacity to absorb and accumulate metals from the environment. Furthermore, humid conditions favor the decomposition of organic substances in the soil, which may result in the release of Cd and its translocation to plants [25]. Previous studies have evaluated whether rainfall may affect the amount of metal present in mushrooms. It has been demonstrated that irrigating spinach and kale with wastewater and rainwater led to an increase in Cd content in the plants [26]. Example data examining seasonal variability in other plants, namely *T. capensis* and *H. psittacorum*, also showed that Cd concentration in the shoots significantly increased over time [27].

The findings from the study suggest that in the last harvest period of *Amanita muscaria*, the average concentration of Pb was approximately at the permissible level for this element in dietary supplements. It is important to note that this is the main harvesting period for *Amanita muscaria* when the highest quantities of these mushrooms can be collected. Elevated blood Pb levels may increase the likelihood of chronic pain. Adults in the USA whose blood Pb levels are in the highest quartile demonstrate a 32% greater risk of chronic pain compared to those with the lowest levels of this metal [28]. Furthermore, studies suggest that elevated blood Pb levels are associated with an increased risk of insomnia, particularly among shift workers [29]. There is also documented evidence linking high Pb levels to symptoms of depression, especially in younger adult males [30]. Consuming *Amanita muscaria* may increase the risk of exposure to Pb, which in turn could worsen mental health conditions. Individuals attempting to self-medicate by consuming *Amanita muscaria* may be at risk for high Pb concentrations in their bodies, thereby exacerbating existing health problems. Thus, the consumption of *Amanita muscaria* may lead to additional health risks associated with high Pb concentrations in these mushrooms. The results obtained indicated a multiple increase in the concentration of Cd in *Amanita muscaria* samples compared to the established maximum allowable level in dietary supplements. Previous studies suggest that chronic exposure to this metal can lead to pain-related behaviors and changes in bone structure, as well as induce depression and anxiety, affecting neurotransmitter levels [31,32]. Other studies have shown that short-term exposure to Cd induces symptoms similar to depression, anxiety, memory disturbances, and biochemical changes in rats, suggesting a potential link between Cd and neurological disorders [33]. Exposure to Cd may also be associated with chronic musculoskeletal pain [34]. Additionally, there is a relationship between exposure to Cd and the severity of insomnia symptoms due to oxidative stress [35]. As a result, individuals who forgo medication in favor of consuming Amanita Muscaria may expose themselves to additional symptoms that they initially sought to reduce.

A limitation of the conducted study is the lack of measurement of Cd and Pb concentrations in the soil from which the *Amanita muscaria* samples were collected. While the metal content within the mushroom itself was analyzed, the potential influence of soil composition on metal accumulation was not assessed. Future research should focus on analyzing the relationship between the concentrations of Cd and Pb in the soil and the levels of these elements in the *Amanita muscaria* samples growing on it. This would provide a more comprehensive understanding of the biogeochemical processes involved in metal uptake by mushrooms. Additionally, such studies could help identify environmental factors that contribute to the variability of metal concentrations in fungal species, offering valuable insights for environmental monitoring and management. Another limitation is the smaller sample size of *Amanita muscaria* during the various harvest periods of this mushroom. In the present study, the decision to collect samples was guided by the availability of mushrooms in the forest, ensuring that the samples were collected at times when they were present in their natural environment.

## 5. Conclusions

High levels of Cd and potentially dangerous concentrations of Pb present in *Amanita muscaria* pose a considerable threat of toxicity. Consuming this mushroom could result in significant health complications. The risks are especially pronounced if the mushroom is ingested repeatedly or over an extended period. Chronic exposure to these heavy metals may exacerbate the adverse effects. Therefore, regular consumption of *Amanita muscaria* should be regarded as a serious health risk.

## Figures and Tables

**Table 1:** Terms of mineralization of analyzed samples.

Mineralization Step	Temperature (°C)	Time [min]
I	150	10
II	160	5
III	180	5
IV	190	15

**Table 2:** Results of the precision and accuracy of the method.

Element	Concentration [Arithmetic Mean]	SD	RSD [%]	Declared Content	Analyte Recovery [%]
Pb ^a^	2.35	0.02	0.85	2.16	109
Cd ^b^	248.75	25.0	10.05	199	125

^a^ mg/kg; ^b^ µg/kg.

**Table 3:** Relationship between the passage of time and the concentrations of Pb and Cd in the collected *Amanita muscaria* samples.

Element	Harvest Time		
	In General	Without Cap Skin	With Cap Skin
Pb	r_s_ = 0.82; *p* < 0.001	r_s_ = 0.94; *p* < 0.001	r_s_ = 0.75; *p* < 0.001
Cd	r_s_ = 0.39; *p* = 0.01	r_s_ = 0.35; *p* = 0.18	r_s_ = 0.41; *p* = 0.03

**Table 4:** Pb and Cd concentrations in the collected *Amanita muscaria* samples with and without cap skin (M—Mean, Q1—First Quartile, Me—Median, Q3—Third Quartile).

Element		Cap Skin	M	Q1	Me	Q3	Statistical Test Result *
Pb	25 August 2023	−	2.03	1.91	2.04	2.13	U = 7; *p* = 0.29
		+	2.15	2.03	2.15	2.19	
	10 September 2023	−	2.57	2.49	2.57	2.64	U = 14; *p* = 0.06
		+	2.44	2.31	2.44	2.54	
	13 October 2023	−	3.13	3.06	3.16	3.19	U = 30; *p* = 0.76
		+	2.9	2.62	2.9	3.27	
Cd	25 August 2023	−	3.29	1.77	2.84	5.26	U = 6.5; *p* = 0.39
		+	6.02	3.05	4.39	7.83	
	10 September 2023	−	6.95	4.14	7.5	9.96	U = 24.5; *p* = 0.39
		+	5.75	4.44	5.49	8.42	
	13 October 2023	−	7.75	2.85	7.9	10.93	U = 28; *p* = 0.62
		+	10.07	4.48	8.7	16.76	

* Mann-Whitney U test.

**Table 5:** Results of the statistical test comparing the average concentrations of Cd and Pb in the collected *Amanita muscaria* samples to the permissible concentrations of these elements in dietary supplements (M—Mean, Q1—First Quartile, Me—Median, Q3—Third Quartile).

Element		M	Q1	Me	Q3	Statistical Test Result *
Pb	25 August 2023	2.1	1.96	2.09	2.25	t(9) = 19.94; *p* < 0.001
	10 September 2023	2.49	2.39	2.49	2.61	t(16) = 13.81; *p* < 0.001
	13 October 2023	2.98	2.71	3.14	3.24	t(16) = 0.2; *p* = 0.85
Cd	25 August 2023	4.93	2.48	3.63	5.12	t(9) = 5.6; *p* = 0.001
	10 September 2023	6.18	4.76	5.49	9.46	t(16) = 6.39; *p* < 0.001
	13 October 2023	9.25	3.67	8.7	15.63	t(16) = 5.73; *p* < 0.001

* Student’s *t*-test for 1 sample.

## Data Availability

The data that support the findings of this study are available from the corresponding authors upon reasonable request.
